# Low Urinary Iodine Concentration Is Associated with Increased Risk for Elevated Plasma Glucose in Females: An Analysis of NHANES 2011–12

**DOI:** 10.3390/nu13124523

**Published:** 2021-12-17

**Authors:** Chisom B. Ezemaduka Okoli, Henok G. Woldu, Catherine A. Peterson

**Affiliations:** 1 Department of Foods & Nutrition, University of Georgia, Athens, GA 30602, USA; chisom.ezemaduka25@uga.edu; 2 Department of Health Management and Informatics, School of Medicine, University of Missouri, Columbia, MO 65211, USA; wolduh@health.missouri.edu; 3 Department of Nutrition and Exercise Physiology, University of Missouri, Columbia, MO 65211, USA

**Keywords:** iodine, iodine status, UIC, insulin resistance, metabolic syndrome

## Abstract

Iodine intake in the US has declined in recent years. Iodine insufficiency increases the risk for inadequate thyroid hormone production and there is growing evidence that sub-clinical hypothyroidism may be disruptive to metabolic health, including insulin resistance (IR). We investigated the association between urinary iodine concentrations (UIC), a measurement of iodine status, and IR in adults. Data from 1286 US adults (≥20 years) in the NHANES 2011–2012 were analyzed. Two subgroups (low = UIC < 100 µg/L and normal = UIC ≥ 100 µg/L) were compared for markers of IR, including fasting plasma glucose (FPG) and insulin, homeostatic model assessment of insulin resistance (HOMA-IR), and glycated hemoglobin (HbA1C). Chi-square test, both linear and logistic regression models were used. In males, there were no significant associations between UIC and markers of IR; however, females with normal UIC had greater risks for elevated HOMA-IR (AOR = 0.56, 95% CI= 0.32–0.99) and HbA1C (AOR = 0.56, 95% CI = 0.34–0.90), while females with low UIC had a greater risk for FPG ≥ 5.6 mmol/L (AOR = 1.73, 95% CI = 1.09–2.72). Results only partially support our hypothesis that UIC is associated with the odds of IR in adults. The finding of an increased risk for elevated FPG, a marker of prediabetes, in female adults with low iodine status requires further investigation.

## 1. Introduction

The Institute of Medicine (IOM) recommends a dietary iodine intake of 150 ug/day for adults based on adequate thyroid iodine accumulation to support thyroid hormone (TH) production [1]. In recent years, iodine intake within the U.S. population has declined from 250 ug/day to 157 ug/day, a trend that mirrors the decrease in sales of iodized salt (from 70% to 53%) [2]. The speculative reasons for this decline are replacement of home-prepared foods with commercially-prepared foods made with non-iodized salts and increased preferences for other salt types for cooking which are non-iodized [3]. All of these have raised concerns about potentially inadequate intakes of iodine despite high intakes of salt from processed foods [4]. 

Classical iodine deficiency is a well-documented health problem. The World Health Organization (WHO) rates it among the top three micronutrient deficiencies worldwide [5]. A consequence of deficient iodine intake is hypothyroidism and if not corrected, is associated with goiter, reproductive damage, fetal and infant mortality, and neurologic defects [6]. Although there are currently limited cases of iodine deficiency in form of goiter in the U.S population, there is growing evidence that sub-clinical hypothyroidism (SCH) may give rise to outcomes disruptive to metabolic health as any little iodine insufficiency increases the risk of inadequate thyroid hormone production [7]. This has been associated with cardiovascular disease risk as well as development of breast cancer [8,9]. More recently, however, evidence has been presented for a connection between SCH and certain conditions related to metabolic syndrome (MetS) such as abdominal obesity, elevated blood lipids, and insulin resistance (IR) [10,11,12,13]. The biological plausibility for this connection lies in the evidence that iodine, through the action of TH, modulates numerous metabolic processes, including thermogenesis, lipid transport and metabolism, and glucose homeostasis [14,15,16]. 

Considering that the prevalence of both iodine insufficiency [17] and MetS [18] remain high among certain groups in the US, further exploration of the association between these two states is warranted. Although several measures are available, urinary iodine concentration (UIC) is a well-accepted, cost-efficient, and easily-obtainable indicator of iodine status [19]. A few studies have used NHANES data to show associations between both TH status and iodine status in the context of dyslipidemia [20,21,22] and there is one published investigation examining the link between TH and IR [7]. However, there is no study to the best of our knowledge that has assessed the association directly between iodine status, as measured by UIC, and IR. The purpose of the study described herein was to investigate the association between UIC and IR in US adults using data from the NHANES 2011–2012 cycle. Our objectives were to: identify socioeconomic and lifestyle variables affecting UIC and markers of IR (fasting glucose, insulin, HOMA-IR, HbA1C); determine the association of UIC with markers of IR; and estimate the risks for IR by UIC. We hypothesized that iodine status, as assessed by UIC, is associated with the odds of IR.

## 2. Materials and Methods

### 2.1. Data Source and Study Sample

NHANES is a cross-sectional examination survey conducted by the National Center for Health Statistics (NCHS). NHANES is based on a complex, stratified, multistage and probability cluster designed to obtain nationally-representative samples of civilian, noninstitutionalized residents in the US [23]. NHANES consists of interviews, laboratory tests, and physical examinations administered by highly trained staff. The protocols used for data in NHANES are approved by the NCHS Research Ethics Review Board and all subjects ≥ 18 years of age give informed consent and participate voluntarily. Detailed descriptions of survey plan and design have been previously provided in the NHANES analytic guidelines [24].

**Figure 1:**
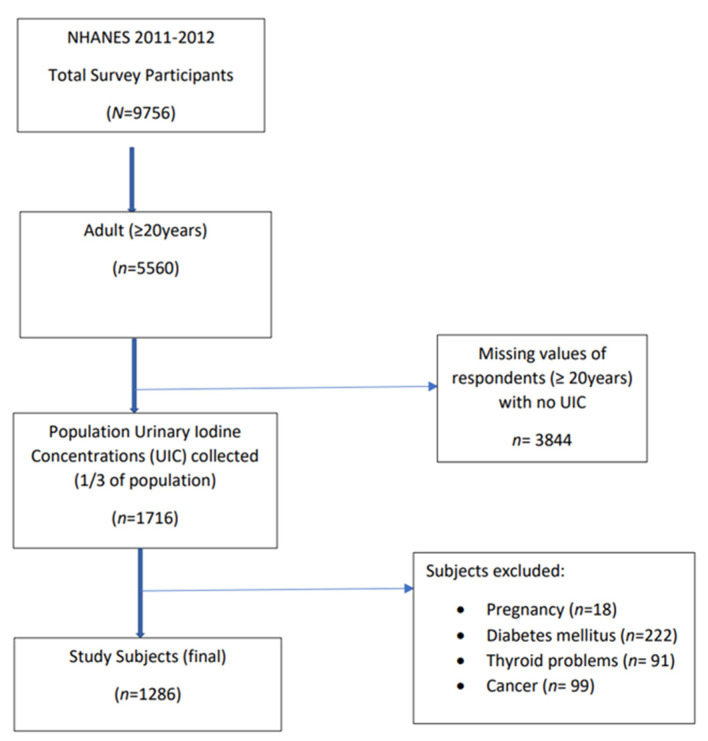
Flow diagram of subject inclusion. Data from the NHANES 2011–2012 cycle was used [25]. Analysis was restricted to adults >20 years of age with urinary iodine concentration (UIC) assessments. Exclusion criteria included thyroid disorders, cancer, diabetes, and pregnancy. The final sample size included 1286 participants.

In this study, data from the NHANES 2011–2012 cycle was used [25]. Analysis was restricted to adult participants >20 years of age who had UIC assessments. Exclusion criteria were those with self-reported history of thyroid disorders, cancer, diabetes, and current pregnancy. After applying exclusion criteria and removing those with missing data, the final sample size included 1286 participants. See Figure 1 for the flow diagram on subject inclusion.

### 2.2. Iodine Status 

One-third of the sample population aged 6 years and older were selected for UIC measurement to represent the U.S. population in NHANES 2011–2012. Spot urine specimens were collected from selected participants and assessed using an inductively-coupled plasma mass spectrometer with dynamic reaction cell technology (ELAN DRC II) (PerkinElmer, Norwalk, CT) [26]. To examine the association of UIC and IR, participants were divided into two groups by UIC (Low UIC < 100 µg/L; Normal UIC ≥ 100 µg/L) according to the classification of population iodine status as defined by WHO [27]. 

### 2.3. Markers of Insulin Resistance

Participants had their blood drawn in the morning after a 9 h fast. Fasting plasma glucose (FPG) and fasting insulin were measured in a subsample (half sample) of persons 12 years and older (*n* = 2881) [28]. Blood specimens were processed, stored, and shipped to Fairview Medical Center Laboratory at the University of Minnesota, Minneapolis, Minnesota for analysis. FPG was analyzed using a hexokinase enzymatic reference method. Insulin was measured using the Elecsys 2010 insulin chemiluminescent “sandwich” immunoassay, which employs two monoclonal antibodies which are specific for human insulin. Using FPG and fasting insulin values, the homeostatic model assessment (HOMA-IR) was calculated and recorded in μU/m as described by Matthews et al. [29].

Glycated hemoglobin A1C (HbA1c), a commonly-used monitor of diabetes control, was measured to determine plasma glucose for the previous 120 days. HbA1c measurements were performed on the A1c G7 HPLC Glycohemoglobin Analyzer (Tosoh Medics, Inc., San Francisco, CA, USA) [30]. The analyzer integrates and reduces the raw data, and then calculates the relative percentages of each hemoglobin fraction. The 2010 Clinical Practice Recommendations were applied in the interpretation (diabetes: HbA1c ≥ 6.5% and pre-diabetes: HbA1c = 5.7%–6.4%) [31].

### 2.4. Covariates

NHANES contains sociodemographic and lifestyle data collected through interviews administered by trained interviewers [24]. Variables included in the statistical analytic models used in this study were: sex (male and female); age (20–39, 40–59, >60 years); race/ ethnicity (non-Hispanic white (NHW), non-Hispanic black (NHB), Mexican American, non-Hispanic Asian (NHA), other Hispanic and Other Races, including multi-racial, grouped into four; NHW, NHB, NHA, and Hispanics (comprises Mexican American, other Hispanics, and other races)); education (less than high school, high school, more than high school); BMI (underweight, <18.5 kg/m^2^; normal, 18.5 to <25 kg/m^2^; overweight, 25 to <30 kg/m^2^, and obese, >30 kg/m^2^); waist circumference (<102 cm and >120 cm (men); <88 cm and >88 cm (women); iodine-containing supplement-use (yes and no); poverty income ratio (low 0 to <1.85, medium 1.85 to <3.5, high ≥ 3.5); smoking (using serum cotinine concentrations) (low <0.015 ng/mL, medium 0.015 ≤ 10 ng/mL, high ≥ 10 ng/mL); alcohol consumption (none, >0 to <1 drink/day, 1 to 2 drinks/day, ≥2 drinks/day); and physical activity (no activity, 0 to <500 MET-min/week, 500 to <1000 MET-min/week, ≥1000 MET-min/week, where MET is the metabolic equivalent of a task). 

### 2.5. Statistical Analyses 

All statistical analyses were conducted using SAS, version 9.4 (SAS Institute, Cary, NC, USA). To account for complex survey design, survey nonresponse, and planned oversampling, we used the SURVEY procedure which includes sample weight, stratum (SDMVSTRA), and primary sampling unit (SDMVPSU) as recommended by NCHS for the NHANES analysis. Chi-square goodness of fit test was performed to investigate the associations between UIC and categorical covariates. Estimates for mUIC with a 95% confidence interval were calculated for covariates. Least significance difference (LSD) was used to test for differences in markers of IR (HOMA-IR, FPG, insulin, HbA1c) by covariates using linear regression (x = covariate; y = marker of IR). Markers of IR were compared between the two UIC groups (i.e., Low vs. Normal) using bivariate analysis. Multiple logistic regression was used to analyze risk for abnormal IR values (FPG ≥ 5.6 mmol/L; insulin >9 μU/mL; HbA1c ≥ 5.7%; HOMA-IR ≥ 2.6) according to the UIC group. Odds ratios (ORs) with 95% confidence intervals (CIs) were calculated in two models for the general population and gender sub-groups (male and female) before (unadjusted) and after controlling for covariates (adjusted). A two-sided alpha level of 0.05 was used to determine statistical significance for all the analyses performed.

## 3. Results

### 3.1. Participant Characteristics by Urinary Iodine Concentration 

Of the 1286 total participants in this analysis, 49 (37.99%) had low UIC, defined as <100 µg/L, and 796 (62.01%) had normal UIC, defined as ≥100 µg/L. The median UIC (mUIC) of the low group was 55.8 µg/L compared to 199.6 µg/L for the normal group. Among the sociodemographic and lifestyle characteristics, only sex and age had statistically significant associations with UIC (Table 1). 

To inform subsequent analyses in this study, the effects of each covariate on mUIC was determined. Only sex (females had lower mUIC), age (40–59 years had lowest mUIC), BMI (normal had lowest mUIC), waist circumference in women (≤88 cm had lower mUIC), and use of iodine-containing supplement (no use had lower mUIC) had significant effects (*p* < 0,05; Table 2).

### 3.2. Analysis of Markers of Insulin Resistance

Unadjusted markers of IR/glucose metabolism according to sociodemographic and lifestyle characteristics are shown in Table 3. Age, race, education, income, smoking, BMI, alcohol use, waist circumference, and physical activity were significantly associated with the majority of the markers of IR. By age, persons ≥60 years old had higher FPG and HbA1c compared with other age groups. Across race, NHB had higher insulin, HbA1c, and HOMA-IR compared with ethnic groups. By education, FPG and HbA1c levels were significantly higher in individuals with education level <high school compared with other education levels. Individuals with low income had higher insulin and HOMA-IR compared with medium- and high-PIR individuals. By smoking, those with high cotinine levels had significantly greater insulin, HbA1c, and HOMA-IR values compared with those with low or medium cotinine levels. Across BMI, obese individuals had higher IR markers compared with BMI <30. Both men and women with greater waist circumference (>102 cm and >88 cm, respectively) had significantly higher markers of IR compared with those within normal waist circumference values. Individuals exercising ≥1000 MET min/week had lower insulin, HbA1c, and HOMA-IR compared with other physical activity groups.

The unadjusted (OR) and adjusted odds ratios (AOR) with 95% CIs for markers of IR by UIC for all participants are described in Table 4. In the unadjusted and adjusted models, no significant differences in risk of elevated markers of IR were found for any of the UIC groups. When the OR analysis was divided by sex, neither the adjusted or unadjusted models were significant in males (data not shown); however, in females, there were several statistical differences. 

Table 6 shows the OR and AOR with 95% CIs for markers of IR by UIC for females only. In the unadjusted model, the odds of elevated HbA1c (≥5.7%) in the low UIC group is 44% less than the normal UIC group. In the adjusted model, the odds of elevated HOMA-IR in adult females with low UIC was 44% less than adult females in the normal UIC group. However, compared with females in the normal UIC group, females with low UIC were more likely to have elevated FPG. 

## 4. Discussion

The objectives of the present study were to use NHANES 2011–2012 data to: (1) identify socioeconomic and lifestyle variables affecting UIC and markers of IR (fasting glucose, insulin, HbA1c, and HOMA-IR); (2) determine the association of UIC with markers of IR; and to (3) estimate the risks of IR by UIC in adults. This analysis showed that the median UIC of adults in the U.S population is above the minimum cut-off for normal iodine status, although there are a few vulnerable groups. Those with significantly lower mUIC were more likely to be female, middle-age, high-income level, normal BMI, and not taking iodine-containing supplements. Our results identifying several socioeconomic and lifestyle factors associated with IR are consistent with the scientific literature (i.e., age, race, education, income, smoking, BMI, alcohol use, waist circumference, and physical activity) [32]. There were no significant associations, unadjusted or adjusted for socioeconomic and lifestyle factors, between measures of IR and UIC for males. This was not the case in females, however, as some of the IR results were significant but conflicting. Females with normal UIC had greater risks for elevated HbA1C and HOMA-IR, while those with low UIC had a greater risk for high FPG. This inconsistency may in part be explained by differences in the diagnostic limitations of each measurement (i.e., HbA1C is a long-term and FPG is a short-term indicator of IR) and/or confounding effects of stronger IR predictors, especially income and body weight status. Therefore, taken together, our results only partially support our hypothesis that UIC is associated with the odds of IR in adults. Our finding of an increased risk for elevated FPG, a marker of prediabetes, in female adults with low iodine status is worthy of further exploration.

While the role of macronutrient intake on MetS and related IR in humans is well-appreciated [33], the effects of micronutrient consumption on IR has yet to be fully recognized. In this analysis, lower iodine status in adult females, but not males, was associated with a greater risk for abnormally high blood glucose levels. This observation is consistent with the research on thyroid hormone and IR. Investigations by Chubb in 2005 [34] and later by Song in 2007 [35] revealed that females with thyroid dysfunction have greater risk for type 2 diabetes (T2D). Animal studies provide some evidence for the mechanism of action showing TH plays a role in glucose uptake by liver and peripheral tissues [36,37]. Further support for this role is demonstrated by Teixeira and colleagues who demonstrated that hypothyroid rats treated with T3 increase glucose uptake through GLUT4 in skeletal muscle [38]. 

Reports on a growing cluster of metabolically-obese, normal-weight (MONW) individuals could explain our findings that women with normal BMI and low UIC are more susceptible to glucose abnormalities [39,40,41,42,43]. For example, one study of 465 healthy volunteers (54% female), designed to describe the prevalence of IR among normal weight, overweight, and obese individuals, found that 16% in the most IR tertile were of normal weight (BMI < 25.0 kg/m2) [44]. In our study, individuals with a normal BMI had lower iodine status compared with overweight or obese. Given the role of iodine in thyroid hormone production, it is tempting to forecast that those with higher BMIs would have lower iodine status based on the ample evidence for compromised thyroid hormone status in the obese [45,46]. The higher iodine status we observed in the overweight and obese is most likely due to excess energy intake, the most common direct cause of overweight/obesity, contributing to incidentally greater intakes of iodine simply because of greater amounts of total food eaten. Conversely, then, leaner participants most likely have lower energy and food intakes. In support of this, Vega-Vega and co-workers recently showed that within a Mexican cohort (a country which also has a national salt iodization program), obese subjects had higher sodium intakes compared with overweight and normal BMI individuals [47]. Furthermore, although adequate iodine availability is essential to the production of thyroid hormone, most of the plausible biological explanations for the relationship between low thyroid hormone status and obesity are related to adipose-derived factors that have direct detrimental effects on the thyroid [45].

Results of some observational studies suggest that different populations may be at greater risk for prediabetes and T2D in the absence of overweight and obesity [48,49]. For example, data from the National Health Interview Survey (NHIS) showed that compared with whites, Asian Americans had a significantly higher risk for T2D despite having markedly lower BMI [50]. Similarly, a recent analysis of NHANES and the Cardiometabolic Risk Reduction in South Asia Surveillance Study (CARRS) records found that Asian-Indians in the normal weight category had 4.6 times greater prevalence of diabetes than white individuals [51]. In our study the disparity in iodine status of non-Hispanic Asians compared with other race groups was unremarkable presumably because of limited statistical power given the small number of participants in this category; however, it was shown to be statistically significant in a report by Herrick et al. in which they noted that the high soy consumption among non-Hispanic Asians was associated with low mUIC [52]. Compared to cow’s milk, soymilk does not contain high amounts of iodine [53]. Soymilk and soy products also contain goitrogens that block the uptake of iodine by the thyroid [54,55]. Therefore, considering iodine’s role via thyroid hormone in thermogenesis and metabolism, it may be speculated that iodine insufficiency-induced SCH could be a contributor predisposing one to metabolic abnormalities like prediabetes. Thus, including an assessment of iodine status in the treatment plan of those normal-body weight patients presenting with symptoms of prediabetes seems prudent, especially in women of Asian ethnicity. 

Despite public measures like salt iodization, iodine status remains suboptimal in a large proportion of women [56]. Our analysis confirmed these observations. Possible contributors to this include limited availability of iodine-containing supplements in the marketplace and poor adherence. Unlike vitamin D (another nutrient deficiency historically considered to be a public health problem), many multi-vitamin/mineral preparations do not contain iodine. However, there has been a modest increase from 51% in 2009 to 61% in 2017 of iodine containing supplements sold in the U.S population [52]. In our study, only a small proportion of the U.S population was found to be taking iodine-containing supplements; those who do had significantly higher mUIC compared with individuals who did not. This observation is supported by several other studies in the literature [57,58,59,60].

Another possible contributing factor to the poorer iodine status of women is the limited knowledge of the health consequences of iodine deficiency among healthcare providers [61]. A study by DeLeo et al. revealed that within 199 midwives and 277 obstetricians studied, 75% of U.S obstetricians and midwives do not recommend an adequate amount of iodine during preconception [62]. This could promote an increase in SCH among women as well as iodine insufficiency-associated consequences. 

Notwithstanding, our present study also showed unexpected decreased risks for elevated HOMA-IR and HbA1c in female adults with UIC < 100 μg/L. These observations could be explained by the presence of shared but conflicting environmental and lifestyle factors associated with iodine status and IR. As an example, excess body fat gain, particularly in the deep abdominal area, is known to be associated with an increase in insulinemia and glucose intolerance [63]. In our study, the majority of female respondents with BMI ≥ 25 kg/m^2^ and waist circumference >88 cm not only had higher levels of each of the IR measurements, but they also had higher iodine status. As previously stated, this is most likely related to greater food/energy intake and thus more possible iodine intake opportunities. This calls for more research using experimental designs that control for these confounders. Furthermore, the differences in the results between the UIC groups may be due to the diagnostic limitation of HbA1c, a long-term reflection of average blood glucose over the past 2 to 3-months compared to FPG, which is a short-term reflection of recent carbohydrate intake and insulin response [64].

This study has both strengths and limitations. We used data from NHANES, a nationally-representative, standardized survey which ensures that results are generalizable and have a high level of validity. This analysis also included the most recent estimate of mUIC for the US population. A study limitation is the well-known variability in UIC which can be highly variable from day-to-day and represent recent rather than usual iodine intake [65]. To overcome this recognized limitation, we used the grouping approach in our analysis. Lastly, due to the nature of a cross-sectional survey, it was not possible to determine a causal relationship between iodine status and IR. However, our finding that females with low UIC, especially of normal BMI, had an increased risk of clinically-elevated blood glucose concentrations is interesting and provides impetus for future investigations to elucidate this association.

## 5. Conclusions

In conclusion, although the mUIC of adults in the US population falls within the range of adequate iodine status, continuous monitoring of certain groups is warranted as there appears to be a decline in iodine status in the general population over the past decade. Of most concern in this study is the possible increase risk for pre-diabetes in women with UIC < 100 µg/L as evidenced by greater FPG, especially among those in middle age with normal BMI. However, more research is needed to confirm a causal relationship between iodine status and IR. 

## Figures and Tables

**Table 1:** Sociodemographic and lifestyle characteristics of study subjects, NHANES 2011–2012 ^1^, overall and by urinary iodine concentration ^1^.

Characteristic	Total		Low UIC (<100 µg/L)		Normal UIC (≥100 µg/L)		Chi-Square
	*n*	*wt’d*%	*n*	*wt’d*%	*n*	*wt’d*%	^2^*p*-Value
All	1286	100	490	37.99	796	62.01	
Sex							
Male	666	50.62	235	16.40	431	34.22	0.0015 **
Female	620	49.38	255	21.59	365	27.79	
Age							
20–39 years	579	44.62	235	17.48	344	27.14	0.0160 *
40–59 years	424	38.58	175	16.38	249	22.20	
≥60 years	283	16.80	80	4.13	203	12.67	
Race							
NHW	429	63.21	164	24.06	265	39.15	0.9865
NHB	338	12.15	128	4.53	210	7.62	
NHA	221	5.80	86	2.27	135	3.53	
Hispanics	298	18.83	112	7.13	186	11.70	
Education							
<High School	278	15.74	103	5.87	175	9.87	0.1321
High School	248	18.91	92	6.14	156	12.77	
>High School	760	65.35	295	25.99	465	39.37	
Poverty Income Ratio ^3^							
Low	670	41.08	244	14.31	426	26.77	0.3670
Medium	250	21.84	100	8.71	150	13.13	
High	366	37.08	146	14.97	220	22.11	
Smoking ^4^							
Low	410	37.63	149	13.75	261	23.88	0.3520
Medium	595	40.11	230	14.68	365	25.43	
High	281	22.26	111	9.56	170	12.70	
BMI ^5^							
Underweight	46	3.08	22	1.32	24	1.76	0.3296
Normal	418	32.32	173	13.61	245	18.70	
Overweight	412	33.10	151	11.52	261	21.58	
Obese	410	31.50	144	11.54	266	19.96	
Supplement ^6^							
Yes	217	18.03	68	5.68	149	12.36	0.1056
No	1069	81.97	422	32.31	647	49.65	
Alcohol							
None	486	31.27	176	10.94	310	20.32	0.1274
>0 to 1 drinks/day	263	21.34	102	8.34	161	13.01	
>1 to 2 drinks/day	211	19.31	90	9.27	121	10.05	
>2 drinks/day	326	28.08	122	9.44	204	18.63	
Physical Activity ^7^							
None	745	51.64	276	19.93	469	31.71	0.8763
0 to <500	249	22.15	97	8.09	152	14.06	
500 to <1000	161	14.09	65	5.61	96	8.48	
≥1000	131	12.12	52	4.35	79	7.77	
Waist Circumference							
Male							
≤102 cm	426	60.85	157	19.75	269	41.10	0.9767
>102 cm	240	39.15	78	12.64	162	26.50	
Female							
≤88 cm	260	43.70	119	20.62	141	23.08	0.1804
>88 cm	360	56.30	136	23.11	224	33.20	

^1^ Data are from the National Health and Nutrition Examination Survey. All data except for sample size are weighted to account for the complex study design according to the guidelines of the National Center for Health Statistics. Values are reported as *n* (weighted percentage). Total of percentages may exceed 100 due to rounding. ^2^ *p* value obtained from the Wald chi-square test (* *p* < 0.05, ** *p* < 0.01). ^3^ PIR, family poverty-income ratio (low: 0–1.85; medium: 1.85< to 3.5; high: >3.5). ^4^ Smoking status defined by a serum cotinine concentration (low: <0.015 mg/L; medium: 0.015 to <10 mg/L; high: ≥10 mg/L). ^5^ BMI: Underweight: <18.5 kg/m^2^; normal weight: 18.5 to >25 kg/m^2^; overweight: 25 to <30 kg/m^2^; and obese: ≥30 kg/m^2^. ^6^ Reported taking supplement containing iodine within the past 30 days. ^7^ Calculated as total MET (metabolic equivalent task minutes)-min/week from self-reported leisure-time physical activities. UIC, urinary iodine concentration; NHW, non-Hispanic white; NHB, non-Hispanic black.

**Table 2:** Median UIC (µg/L) of US adults by demographic and lifestyle characteristics, NHANES 2011–2012 ^1^.

Characteristic		mUIC µg/L	(95% CI)
	Overall	126.6	(111.9, 141.2)
Sex	Male	145.6	(124.1, 167.1)
	Female	111.2 *	(96.4, 126.0)
Age	20–39 years	120.1	(98.1, 142.1)
	40–59 years	113.9 *	(94.6, 133.3)
	≥60 years	157.3	(133.8, 180.9)
Race	NHW	120.2	(98.9, 141.4)
	NHB	135.1	(109.8, 160.4)
	NHA	128.9	(114.0, 143.8)
	Hispanics	136.1	(113.9, 158.2)
Education	<High School	136.4	(113.4, 159.5)
	High School	127.8	(96.6, 159.1)
	>High School	121.4	(103.3, 139.5)
PIR ^2^	Low	141.0	(125.9, 156.1)
	Medium	120.4	(97.7, 143.1)
	High	117.1 *	(97.7, 136.6)
Smoking ^3^	Low	129.9	(106.7, 153.1)
	Medium	130.1	(108.1, 151.9)
	High	113.8	(90.9, 136.8)
BMI ^4^	Underweight	118.6	(71.5, 165.6)
	Normal	111.0 *	(95.4, 126.6)
	Overweight	138.8	(115.5, 162.1)
	Obese	139.2	(115.3, 163.1)
Supplement ^5^	Yes	148.4	(124.5, 172.3)
	No	121.8 *	(106.7, 136.9)
Alcohol	None	133.7	(101.0, 166.5)
	<0 to 1 drinks/d	121.3	(96.3, 146.3)
	>1 to 2 drinks/d	101.0	(72.1, 129.9)
	>2 drinks/d	140.3	(121.1, 159.4)
Physical Activity ^6^	No Activity	129.8	(107.7, 151.6)
	0 to <500	119.5	(87.2, 151.8)
	500 to <1000	119.1	(95.3, 142.8)
	≥1000	138.7	(113.3, 163.9)
Waist Circumference	Male		
	≤102 cm	146.6	(124.0, 169.2)
	>102 cm	143.6	(114.0, 173.2)
	Female		
	≤88 cm	104.1 *	(87.3, 120.8)
	>88 cm	116.8	(97.9, 135.7)

^1^ Data are from the National Health and Nutrition Examination Survey; * indicate statistically-significant category within characteristic, *p* < 0.05. ^2^ PIR, family poverty-income ratio (low: 0–1.85; medium: 1.85< to 3.5; high: >3.5). ^3^ Smoking status defined by a serum cotinine concentration (low: <0.015 mg/L; medium: 0.015 to <10 mg/L; high: ≥10 mg/L). ^4^ BMI: Underweight: <18.5 kg/m^2^; normal weight: 18.5 to >25 kg/m^2^; overweight: 25 to <30 kg/m^2^; and obese: ≥30 kg/m^2^. ^5^ Reported taking supplement containing iodine within the past 30 days. ^6^ Calculated as total MET (metabolic equivalent task minutes)-min/week from self-reported leisure-time physical activities. UIC, urinary iodine concentration; NHW, non-Hispanic white; NHB, non-Hispanic black.

**Table 3:** Unadjusted insulin resistance/glucose metabolism biomarkers by covariates categories for US adults, NHANES 2011–2012 ^1^.

		FPG	Insulin	Hb1Ac	HOMA- IR
Characteristic		mmol/L	μU/mL	%	
Sex	Male	5.7 ± 0.07	13.5 ± 0.70	5.5 ± 0.03	3.5 ± 0.20
					
	Female	5.4 ± 0.07	12.0 ± 0.70	5.4 ± 0.03	2.9 ± 0.13
	r^2^, %	<1	<1	<1	<1
Age	20–39 years	5.4 ± 0.05 *	12.7 ± 0.60	5.3 ± 0.03 **	3.1 ± 0.16
	40–59 years	5.6 ± 0.10	13.3 ± 1.02	5.5 ± 0.03	3.5 ± 0.31
	≥60 years	5.7 ± 0.15	11.6 ± 1.15	5.7 ± 0.06	3.1 ± 0.30
	r^2^, %	2.8	<1	7	<1
Race	NHW	5.5 ± 0.04	12.4 ± 0.68	5.4 ± 0.03	3.1 ± 0.17
	NHB	5.5 ± 0.08	15.4 ± 0.52 **	5.6 ± 0.02 **	3.9 ± 0.13 **
	NHA	5.4 ± 0.09	12.2 ± 1.21	5.5 ± 0.06	3.0 ± 0.35
	Hispanics	5.6 ± 0.09	12.9 ± 1.39	5.5 ± 0.03	3.4 ± 0.39
	r^2^, %	<1	<1	1.3	<1
Education	<High School	5.8 ± 0.11 **	13.9 ± 1.20	5.6 ± 0.03 **	3.7 ± 0.30
	High School	5.5 ± 0.06	13.6 ± 0.90	5.4 ± 0.03	3.4 ± 0.25
	>High School	5.4 ± 0.04	12.2 ± 0.60	5.4 ± 0.03	3.0 ± 0.15
	r^2^, %	1.5	<1	2.5	1
PIR ^2^	Low	5.5 ± 0.07	14.4 ± 0.79 *	5.5 ± 0.03 *	3.6 ± 0.24 *
	Medium	5.6 ± 0.06	12.3 ± 0.88	5.4 ± 0.04	3.2 ± 0.27
	High	5.5 ± 0.08	10.9 ± 0.87	5.4 ± 0.04	2.7 ± 0.21
	r^2^, %	<1	2.5	<1	2
Smoking ^3^	Low	5.5 ± 0.07	11.5 ± 0.73	5.4 ± 0.03	2.9 ± 0.17
	Medium	5.6 ± 0.09	12.9 ± 0.91	5.5 ± 0.03	3.3 ± 0.25
	High	5.6 ± 0.05	13.9 ± 0.77 *	5.5 ± 0.02 *	3.5 ± 0.18 *
	r^2^, %	<1	<1	<1	<1
BMI ^4^	Underweight	5.3 ± 0.11	16.7 ± 5.16	5.3 ± 0.05	4.1 ± 1.32
	Normal	5.3 ± 0.08	8.3 ± 0.68	5.3 ± 0.04	2.0 ± 0.16
	Overweight	5.5 ± 0.06	11.3 ± 0.80	5.4 ± 0.03	2.8 ± 0.19
	Obese	5.7 ± 0.07 **	18.3 ± 1.00 **	5.6 ± 0.04 **	4.8 ± 0.27 **
	r^2^, %	3.1	18	3.8	18.2
Supplement ^5^	Yes	5.5 ± 0.07	12.7 ± 1.53	5.4 ± 0.03	3.2 ± 0.43
	No	5.6 ± 0.04	12.8 ± 0.53	5.4 ± 0.03	3.2 ± 0.13
	r^2^, %	<1	<1	<1	<1
Alcohol	None	5.4 ± 0.06 *	13.1 ± 1.01	5.5 ± 0.03	3.3 ± 0.26
	>0 to 1 drinks/d	5.5 ± 0.15	10.5 ± 0.82 **	5.5 ± 0.06	2.6 ± 0.22 **
	>1 to 2 drinks/d	5.7 ± 0.11	13.2 ± 1.44	5.3 ± 0.04	3.5 ± 0.42
	≥2 drinks/d	5.6 ± 0.06	13.7 ± 0.59	5.4 ± 0.03	3.5 ± 0.14
	r^2^, %	1.3	1.4	1.1	1.5
Physical Activity ^6^	No activity	5.5 ± 0.03	14.3 ± 0.58	5.5 ± 0.03	3.6 ± 0.10
	0 to <500	5.6 ± 0.13	12.5 ± 1.02	5.4 ± 0.05	3.2 ± 0.30
	500 to <1000	5.5 ± 0.08	11.0 ± 0.83	5.4 ± 0.05	2.8 ± 0.20
	≥1000	5.4 ± 0.09	9.6 ± 1.22 **	5.3 ± 0.04 **	2.4 ± 0.30 **
	r^2^, %	<1	3.3	4.7	2.9
Waist Circumference	Male				
	≤102 cm	5.6 ± 0.07	10.3 ± 0.70	5.4 ± 0.02	2.6 ± 0.18
	>102 cm	5.8 ± 0.08 **	18.3 ± 1.30 **	5.6 ± 0.04 **	4.9 ± 0.39 **
	r^2^, %	1.1	14.8	4.4	14.2
	Female				
	≤88 cm	5.1 ± 0.05	9.1 ± 1.21	5.3 ± 0.03	2.1 ± 0.28
	>88 cm	5.6 ± 0.12 **	14.0 ± 0.61 **	5.5 ± 0.04 **	3.5 ± 0.12 **
	r^2^, %	6	6.9	5	8.7

^1^ Data are from the National Health and Nutrition Examination Survey, * represent Least significant differences (LSD) obtained from bivariate analysis in a linear regression test (* *p* <0.05, ** *p*< 0.01). ^2^ PIR, family poverty-income ratio (low: 0–1.85; medium: 1.85< to 3.5; high: >3.5). ^3^ Smoking status defined by a serum cotinine concentration (low: <0.015 mg/L; medium: 0.015 to <10 mg/L; high: ≥10 mg/L). ^4^ BMI: Underweight: <18.5 kg/m^2^; normal weight: 18.5 to >25 kg/m^2^; overweight: 25 to <30 kg/m^2^; and obese: ≥30 kg/m^2^. ^5^ Reported taking supplement containing iodine within the past 30 days.^6^ Calculated as total MET (metabolic equivalent task minutes)-min/week from self-reported leisure-time physical activities. UIC, urinary iodine concentration; NHW, non-Hispanic white; NHB, non-Hispanic black.

**Table 4:** Prevalence of elevated markers insulin resistance in relation to urinary iodine concentration in US adults, NHANES 2011–2012 ^1^.

Elevated IR Marker	Model	Low UIC (<100 µg/L)	Normal UIC (≥100 µg/L)	^2^*p*-Value
		OR (95% CI)	Referent	
FPG ≥ 5.6 mmol/L	1	1.08 (0.72–1.62)	1	0.7036
	2	1.11 (0.77–1.59)	1	0.5630
Insulin > 9 μU/mL	1	0.99 (0.60–1.65)	1	0.1877
	2	0.99 (0.59–1.67)	1	0.9926
HOMA-IR ≥ 2.6	1	0.86 (0.55–1.34)	1	0.4744
	2	0.83 (0.56–1.25)	1	0.3489
HbA1c ≥ 5.7%	1	0.83 (0.57–1.21)	1	0.3091
	2	0.91 (0.64–1.28)	1	0.5502

^1^ Data are from the National Health and Nutrition Examination Survey. ^2^ Multiple logistic regression analysis was performed to estimate odds ratio for elevated marker of insulin resistance in female adults from NHANES 2011–2012 in two models: unadjusted (model 1) and adjusted (model 2). See Table 5 for *p* values of the covariates used in model 2. IR, insulin resistance; UIC, urinary iodine concentration; OR, odds ratio; CI, confidence interval; FPG, fasting plasma glucose; HbA1c, glycated hemoglobin A1C; HOMA-IR, homeostatic model assessment of insulin resistance.

**Table 5:** Covariates used in adjusted model (model 2) for elevated markers of insulin resistance in relation to urinary iodine concentration of US female adults, NHANES 2011–2012 ^1^.

Elevated IR Marker	Covariates	*p*-Value
FPG ≥ 5.6 mmol/L	Age	0.0412 *
	Smoking	0.1767
	BMI	0.0810
	Waist circumference	0.0360 *
Insulin >9 μU/mL	Race	0.4766
	Smoking	0.0996
	BMI	0.8079
	Waist circumference	0.0496
HOMA-IR ≥ 2.6	Smoking	0.2743
	Alcohol Use	0.1018
	BMI	0.0003 **
	Waist circumference	0.0012 **
	Iodine supplement use	0.8537
HbA1C ≥ 5.7%	Age	0.0006 **
	Education	0.9270
	BMI	0.0022 **
	Waist circumference	0.0205 *
	Physical activity	0.4364

^1^ Covariates were statistically significant in bivariate logistic regression for each IR marker and thus, used in the adjusted model analysis (model 2 of Table 5 and Table 6), (* *p* < 0.05, ** *p* < 0.01). IR, insulin resistance; FPG, fasting plasma glucose; HbA1c, glycated hemoglobin A1C; HOMA-IR, homeostatic model assessment of insulin resistance.

**Table 6:** Prevalence of elevated markers insulin resistance in relation to urinary iodine concentration in US female adults, NHANES 2011–2012 ^1^.

Elevated IR Marker	Model	Low UIC (<100 µg/L)	Normal UIC (≥100 µg/L)	^2^*p*-Value
		OR (95% CI)	Referent	
FPG ≥ 5.6 mmol/L	1	1.52 (0.94–2.44)	1	0.0839
	2	1.73 (1.09–2.72)	1	0.0211 *
Insulin > 9 μU/mL	1	1.04 (0.58–1.87)	1	0.8873
	2	1.08 (0.54–2.16)	1	0.8120
HOMA-IR ≥ 2.6	1	0.91 (0.46–1.80)	1	0.7734
	2	0.56 (0.32–0.99)	1	0.0478 *
HbA1c ≥ 5.7%	1	0.56 (0.34–0.90)	1	0.0208 *
	2	0.58 (0.34–1.02)	1	0.0563

^1^ Data are from the National Health and Nutrition Examination Survey. ^2^ Multiple logistic regression analysis was performed to estimate odds ratio for elevated marker of insulin resistance in female adults from NHANES 2011–2012 in two models: unadjusted (model 1) and adjusted (model 2). (* *p* < 0.05). See Table 5 for *p* values of the covariates used in model 2. IR, insulin resistance; UIC, urinary iodine concentration; OR, odds ratio; CI, confidence interval; FPG, fasting plasma glucose; HbA1c, glycated hemoglobin A1C; HOMA-IR, homeostatic model assessment of insulin resistance. The *p*-values for covariates controlled for in the adjusted model are shown for each marker of IR in adult females are shown in Table 5.

## Data Availability

Publicly-available datasets were analyzed in this study. Data can be found at this website: https://wwwn.cdc.gov/nchs/nhanes/ (accessed on 23 November 2021).
